# Perceptual Health and Wellbeing, Self-Reported Sleep, and Hydration Status in Youth Soccer Players During Competition

**DOI:** 10.1155/2024/5086660

**Published:** 2024-10-30

**Authors:** Michael King, Rachel Kimble, Matthew Brown, Seamus McCafferty, Hannah Lithgow

**Affiliations:** ^1^School of Cardiovascular and Metabolic Health, University of Glasgow, Glasgow, UK; ^2^Playermaker, Division of Sport, Exercise and Health, School of Health and Life Sciences, University of the West of Scotland, Glasgow, UK; ^3^Hampden Sports Clinic, Independent Researcher, London, UK; ^4^Independent Researcher, Glasgow, UK; ^5^Department of Biology, School of Energy, Geoscience, Infrastructure and Society, Heriot-Watt University, Edinburgh, UK

**Keywords:** performance, recovery, sleep, soccer, wellbeing

## Abstract

**Introduction:** The aim of this study was to assess match time courses on hydration, wellness, and sleep as well as the interrelationship between these variables in youth national soccer players.

**Materials and Methods:** Under-17 and under-19 youth national soccer players (age range: 16.96 ± 1.17 years) completed a perceptual wellness questionnaire, self-reported their sleep, and carried out hydration assessments each morning during a period of competitive match play.

**Results:** Players reported having significantly more sleep leading into the day of a match (MD) compared to both the evening after a match (MD-2; *p* < 0.001; CI = 7.972 and 8.212) and the evening before a match (MD-1; *p* < 0.001; CI = 7.996 and 8.174). Furthermore, players reported better health and wellbeing scores on MD compared to both MD-1 (*p* < 0.001; CI = 19.231 and 19.692) and MD-2 (*p* < 0.001; CI = 18.911 and 19.489). When self-reported sleep was correlated with the individual components of health and wellbeing, it was highlighted that there were significant effects for fatigue (*r* = 0.304, *p* < 0.001; CI = 0.250 and 0.383), mood (*r* = 0.170, *p* < 0.001; CI = 0.112 and 0.243), general muscle soreness (*r* = 0.225, *p* < 0.001; CI = 0.162 and 0.306), and stress (*r* = 0.203, *p* < 0.001; CI = 0.147 and 0.274).

**Conclusion:** It is important to consider sleep strategies to mitigate the potential impact of lack of sleep following match play. Self-reported sleep seems to be appropriate for estimating individual components of health and wellbeing, and therefore may be a suitable replacement for perceptual wellness questionnaires.

## 1. Introduction

The physical and technical demands of football association (soccer) have increased in recent years into a much faster, intensive, and more competitive game [1]. Training and competitive loads may represent stressful factors for players. Particularly, tournament match play inherently involves a congested fixture schedule, which commonly involves participating in multiple matches (two to three) within a weekly microcycle, often with as little as 48–72 h recovery between games. Sleep disruption and negative wellbeing have all been reported in response to congested fixtures [2, 3], compromising not only player's preparedness for the following match but also potentially leading to excessive fatigue and increased risk of illness and injury [4–6]. As a result, to monitor athlete's wellbeing and assess preparation and susceptibility to illness and injury, it is commonplace in soccer to assess markers of hydration status and wellness (fatigue, stress, muscle soreness, and sleep perception) during times of intensive training and competition [7–9]. Indeed, professional sporting organizations invest considerable resources in collecting and analyzing data to better understand the factors that influence training and performance. However, the usefulness of such data and its functional relevance has been questioned [10] and often little consideration is given to the collinearity and interrelationships of these variables [11, 12].

The importance of appropriate hydration for supporting players' health and performance is well documented (see [13]). Loss of water during exercise (dehydration), even if not immediately noticeable, negatively impacts performance during both training sessions and match play [8]. Therefore rehydration, particularly with the addition of carbohydrates is an important recovery strategy especially during congested fixtures [14]. Levels of dehydration may also be related to aspects of wellness such as fatigue and mood, as well as cognitive performance [15]. For example, Leão et al. [8] found a correlation between dehydration (measured as percentage body weight loss) and fatigue, stress, and muscle soreness in professional adult soccer players during preseason. Interestingly, they also found a trend (*p*=0.05) for an association between subjective sleep in the same study. The relationship between hydration status and sleep parameters has received very little attention [16], despite some evidence that induced dehydration may be negatively impacting subjective sleep in recreational athletes [17].

As well as hydration, sleep is a significant aspect of a players' health, recovery, and performance, gaining significant research attention over recent years [18, 19]. Sleep quality is a crucial part of the stress-recovery balance and wellbeing of a player [20]. For example, young university athletes who reported < 8 h sleep reported worse perceived training quality, as well as lower mood states and more fatigue [21]. Similarly, wellbeing can also impact training output and physical performance [22, 23], and sleep may mediate negative wellbeing to some extent. However, while the importance of these individual monitoring systems has been highlighted previously, to the best of our knowledge, there is not a study that has reported them collectively preceding tournament match play, and the relationship between these variables in youth football players has not yet been investigated. In this context, the aim of the current study was to assess match time courses on hydration, wellness, and sleep as well as the interrelationship between these variables in youth national soccer players. It is hypothesized that the proximity to match day (MD) will significantly influence hydration status, wellness, and sleep quality. Furthermore, it is hypothesized that there will be a significant interrelationship between these variables, such that the impairment of one variable will be correlated with the impairment of another.

## 2. Materials and Methods

This is a retrospective analysis of an anonymized archived dataset from the Scottish Football Association (SFA), accessed in October 2023 following ethical approval. The study was reviewed and granted ethical approval by the Health and Life Sciences School Ethics Committee (ethical approval no. 16889). The committee waived the requirement for informed consent.

### 2.1. Participants

**Figure 1:**
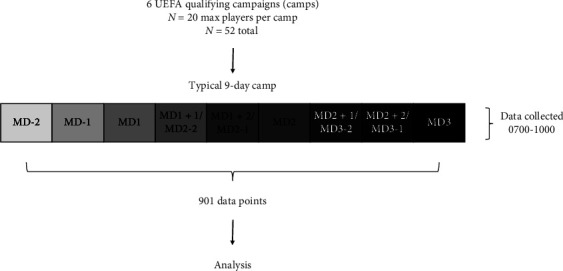
Study design.

Fifty-two different Under-17 and Under-19 national soccer team players (age range: 16.96 ± 1.17) completed assessments of health and wellbeing, self-reported sleep, and hydration status each morning of 6 separate UEFA qualifying campaigns (all ranging from 9 to 10 days in duration) during the period of October 2021 to May 2023. Each squad consisted of 20 players in each camp, and some players were present across multiple camps. A total of 901 data points were collected across each of the camps from a possible 1140 data points (79% of all potential data points were captured). See Figure 1 for the study design. As these assessments were part of daily routine whilst traveling with a national squad, players were familiar with the assessments and therefore no formal familiarization sessions were conducted. If some individuals were completing assessments for the first time, an additional effort was made by practitioners to ensure they understood what was required.

### 2.2. Procedure

The intention of the morning assessments was to examine perceptual fatigue responses, self-reported sleep volume, and hydration status daily. Morning clinics were setup in each camp in a designated medical room in the team hotel. Players would visit the room every morning before breakfast, or any other activities on camp, and were asked to bring with them a hand-held mobile device (smartphone or tablet) and a urine sample that had been taken that morning. If they did not possess their own smart device, a device was made available for them to use. Urine samples were taken by players and contained in a purpose-made urine sample pot.

### 2.3. Health and Wellbeing Assessment

**Figure 2:**
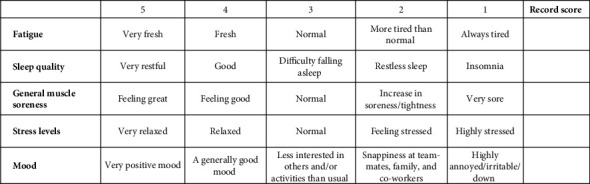
Perceptual wellness questionnaire.

A specially created psychological questionnaire that has been used previously by Dupont et al. [6] in similar settings with athletes was filled out each morning of the camp during the study period. On a five-point scale (scores of 1–5, with one-point increments; see Figure 2), the questionnaire evaluated their levels of mood, stress, general muscle soreness, sleep quality, and fatigue to quantify perceptual wellness. The five scores were then added to get the overall wellbeing score. The questionnaire was designed into a purpose-built Microsoft Form by the practitioners on camp and was made accessible to players via a printed QR code that could be scanned using personal smart devices and would take them directly to the questionnaire. At the end of the questionnaire, players were also asked to self-report how many hours (rounding up) they slept for the previous night (scale of 0 h–12+ hours, with 30-min increments), that is, the volume of self-reported sleep related to the previous night's sleep. For example, on the MD, players were reporting how much sleep they had leading into MD.

### 2.4. Hydration Assessment

Players brought their urine samples with them to a designated medical room in the team hotel where it was provided for medical staff to test using an Osmocheck urine analysis unit (Vitech Scientific Ltd). The Osmocheck unit was used to provide an instant measure of hydration status by displaying a measurement of urine osmolality between 0 and 1500 mOsmols/kgH20. Players were given their urine osmolality score verbally by a member of the medical staff and asked to enter it into the online Microsoft Form at the end of the health and wellbeing questionnaire.

### 2.5. Statistical Analysis

All descriptive statistics were reported as mean ± standard deviation. All data were deemed normally distributed following a Shapiro–Wilk test. One-way ANOVA, followed by post hoc (Tukey) analysis were used to examine for any significant differences between self-reported sleep, hydration status, and perceptual health and wellbeing depending on the proximity to MD. Proximity to MD was reported as MD, the day before MD (MD-1), and 2 days before MD (MD-2). Cohen's d effect sizes were also calculated to determine the magnitude of the difference between self-reported sleep, hydration status, and perceptual health and wellbeing depending on the proximity to MD and were interpreted as 0.2, 0.5, and > 0.8 for small, medium, and large effects, respectively. Correlational analysis (Pearson's *r*) was used to determine any associations between self-reported sleep, hydration status, and perceptual health and wellbeing. During the analysis, health and wellbeing were analyzed as one composite score and separately as its individual components (fatigue, sleep quality, stress, general muscle soreness, and mood). All analysis was conducted using Jamovi (Jamovi, 2021) statistical analysis software. Statistical significance was accepted at *p* < 0.05.

## 3. Results

Inferential statistics were used to determine the difference between the total number of hours of self-reported sleep, hydration status, and perceptual health and wellbeing scores depending on the proximity to the MD. Descriptive data for each metric are shown in Table 1.

### 3.1. Proximity to MD, Self-Reported Sleep, Hydration Status, and Health and Wellbeing

Players reported having significantly more sleep leading into the MD compared to both the evening after a match (MD-2; *p* < 0.001, ES: 0.3) and into the morning before a match (MD-1; *p* < 0.001, ES: 0.4). Furthermore, players reported better health and wellbeing scores on MD compared to both MD-1 (*p* < 0.001, ES: 0.4) and MD-2 (*p* < 0.001, ES: 0.5). Despite there being significant differences regarding how much players slept and how they reported their health and wellbeing, there were no significant effects highlighted for proximity to MD and player hydration status.

### 3.2. Association Between Self-Reported Sleep, Hydration Status, and Health and Wellbeing

Correlational analysis was used to determine associations between self-reported sleep, perceptual health and wellbeing, and hydration status. A significant positive correlation was noted between self-reported sleep and health and wellbeing score (*r* = 0.03, *p* < 0.001), however, sleep was reported as part of the health and wellbeing questionnaire. Moreover, when self-reported sleep was correlated with the individual components of the perceptual health and wellbeing questionnaire, there were significant effects for fatigue (*r* = 0.304, *p* < 0.001), mood (*r* = 0.170, *p* < 0.001), general muscle soreness (*r* = 0.225, *p* < 0.001), and stress (*r* = 0.203, *p* < 0.001).

There were no significant correlations reported between self-reported sleep and hydration status, or perceptual health and wellbeing and hydration status, regardless of whether perceptual health and wellbeing were treated as one composite score or as its individual components.

## 4. Discussion

The aim of the current study was to assess match time courses on hydration, wellness, and sleep as well as the interrelationship between these variables in youth national soccer players. The main findings were that self-reported sleep preceding MD was better than that on the night of the MD or MD-2. Similarly, MD wellbeing was higher than that reported in the days preceding the match, while hydration status did not differ. We also found a positive relationship between self-reported sleep and overall and individual domains of wellbeing.

Overall, the players self-reported more sleep the night before the match, which is consistent with the findings of others and may be due to the lower training demands preceding MDs [24]. This coincided with better wellbeing scores on the morning of MDs. Higher wellbeing on MD has been reported and is suggestive of a conducive training structure attempting to ensure player's preparedness for official competitive games [25]. Therefore, the improved wellbeing on MD may be mediated by better sleep and recovery in the preceding night. Several studies have reported negative effects of sleep deprivation on athlete wellbeing, specifically in relation to mood states [26, 27], while proper sleep (≥ 8 h) may exhibit less additional psychological stress [21]. In addition, sleep may be important to muscle recovery by suppressing hormones related to protein synthesis and upregulating those involved in catabolism [28]. However, to the best of our knowledge, this is the first study to find a positive correlation between simple self-reported sleep duration and the overall subdomains (mood, stress, general muscle soreness, sleep quality, and fatigue) of the athlete wellbeing scale. Given the staff and resources required to run daily collection of athlete data [10] and the potential for data overload [11], these results present a potential opportunity to streamline this process, in that simple self-reported sleep may be a useful indicator of match preparedness. However, caution should be made when interpreting this, as the average sleep duration even on alternative nights was within the recommended limits for this age group [29].

Dehydration may negatively impact wellbeing [8] and sleep [17]. Dehydration can contribute to fatigue in football [30] and has been shown to activate the serotoninergic system which may alter mood states [31]. The hydration status of youth soccer players preceding MDs is not well documented. Contrary to our findings, Silva and Mündel [32] found that the mean hydration status, measured by urine specific gravity, indicated that youth players were dehydrated pretraining on MD-2 and MD-1 in youth Brazilian football players, although this may be attributable to the differences in ambient temperature between the two studies. Below < 700 mOsmol/kg for urine osmolality is indicative of dehydration [33]. As can be seen from the average data, in the current study, most players were dehydrated on the morning of testing, indicating familiarity with proper hydration strategies, at least before exercise. In contrast to these findings, Leão et al. [8] found a negative correlation between percentage dehydration and fatigue, stress, and total wellbeing in soccer players during preseason. Nevertheless, given that players did not appear to be dehydrated in the current study, it is perhaps unsurprising that we did not find a relationship between hydration status, wellbeing, or sleep in the current study.

This study has limitations that warrant discussion. First, the subjective nature of the self-reported measures should be acknowledged, particularly since it is well known that players may answer in a “socially desirable” manner [10]. Second, the data presented are one-off measurements and therefore do not fully capture the wellbeing and hydration status of the player's which can vary throughout the day. Third, player positions, duration with the club, and time of the match are not included. Fourth, a limitation of our study was the inability to conduct a within-subject analysis. This constraint prevented us from calculating within-subject correlations, which would have provided a more precise understanding of the intraindividual variability in self-reported sleep, hydration status, and perceptual health and wellbeing metrics. Instead, we relied on means comparison analysis to identify group differences depending on proximity to MD. While this approach allowed us to analyze the data with the structure available, it may not capture the full complexity of individual-level changes over time, and this should be considered when interpreting the findings. Finally, we cannot exclude the effect of confounding factors such as nutrition and temperature on the measurements.

In summary, the current study suggests that youth football players have adequate hydration and sleep during training and match play in qualifying campaigns. Self-reported sleep was higher the night preceding a match and wellbeing was better on MD morning. Moreover, self-reported sleep was associated with overall wellbeing and may be an easy surrogate marker to collect to inform player readiness; however, further studies are needed to establish clear guidelines on this.

### 4.1. Perspective

Ensuring the wellbeing and preparedness of youth soccer players for competition is paramount as it not only maximizes their performance potential but also safeguards their physical health, promoting long-term athletic development. Our findings not only provide crucial information for optimizing training regimens and performance strategies but also underscore the importance of considering youth athletes' subjective experiences, such as sleep quality and wellbeing, alongside objective measurements, such as hydration status. Our findings also demonstrate that youth players were “ready” for competition; however, whether they believed their responses would feed into coach decision-making is unknown. Therefore, these performance determinants should be considered and discussed in sports, with an emphasis on open and honest communication, thereby fostering a holistic approach to athlete development and maturation.

### 4.2. Practical Applications and Future Research

The insights gained from this study have several practical applications for coaches, sports scientists, and other stakeholders involved in the development of youth soccer players. First, integrating subjective measures such as self-reported sleep quality and wellbeing into regular monitoring routines can provide a more comprehensive understanding of an athlete's readiness for competition. This comprehensive approach allows coaches to tailor training loads, recovery strategies, and match preparations more effectively, promoting both performance enhancement and injury prevention.

Moreover, the findings highlight the need for fostering open communication between athletes and coaches regarding players' perceived readiness and wellbeing. Encouraging athletes to voice their subjective experiences can empower them, making them active participants in their development process and potentially enhancing their overall athletic journey.

Future research should aim to explore the impact of such subjective measures on coaching decisions and performance outcomes in youth sports. Longitudinal studies tracking these variables over extended periods could provide deeper insights into their role in athlete development and their potential to influence long-term success. In addition, further investigation into the integration of objective and subjective data in decision-making processes within youth sports environments could pave the way for more individualized and effective coaching strategies.

Finally, addressing the limitations of the current study, future research should consider designs that enable within-subject analysis, potentially through the collection of more detailed, longitudinal data. This would allow for a more nuanced understanding of how individual variations in sleep, hydration, and wellbeing impact performance and how these factors interact over time.

## Figures and Tables

**Table 1:** Means and standard deviations for hours of sleep, hydration and perceptual health, and wellbeing.

Proximity to match day	Self-reported sleep (hours)	Hydration (mOsmols/kgH20)	Health and wellbeing (arbitrary units)
Match Day −2	8.09 ± 0.92∗	597 ± 220	19.20 ± 2.31∗
Match Day −1	8.08 ± 0.79∗	599 ± 203	19.50 ± 2.13∗
Match Day	8.37 ± 0.72	593 ± 207	20.40 ± 2.13

^∗^Significantly different to match day value.

## Data Availability

The raw data used to support the findings of this study have been deposited in the open science framework repository.
